# A novel framework for the identification of drug target proteins: Combining stacked auto-encoders with a biased support vector machine

**DOI:** 10.1371/journal.pone.0176486

**Published:** 2017-04-28

**Authors:** Qi Wang, YangHe Feng, JinCai Huang, TengJiao Wang, GuangQuan Cheng

**Affiliations:** 1 Science and Technology on Information Systems Engineering Laboratory, National University of Defense Technology, Changsha, Hunan, China; 2 Second Medical Military University, Shanghai, China; UMR-S1134, INSERM, Université Paris Diderot, INTS, FRANCE

## Abstract

The identification of drug target proteins (IDTP) plays a critical role in biometrics. The aim of this study was to retrieve potential drug target proteins (DTPs) from a collected protein dataset, which represents an overwhelming task of great significance. Previously reported methodologies for this task generally employ protein-protein interactive networks but neglect informative biochemical attributes. We formulated a novel framework utilizing biochemical attributes to address this problem. In the framework, a biased support vector machine (BSVM) was combined with the deep embedded representation extracted using a deep learning model, stacked auto-encoders (SAEs). In cases of non-drug target proteins (NDTPs) contaminated by DTPs, the framework is beneficial due to the efficient representation of the SAE and relief of the imbalance effect by the BSVM. The experimental results demonstrated the effectiveness of our framework, and the generalization capability was confirmed via comparisons to other models. This study is the first to exploit a deep learning model for IDTP. In summary, nearly 23% of the NDTPs were predicted as likely DTPs, which are awaiting further verification based on biomedical experiments.

## Introduction

In the domain of drug development, the identification of drug target proteins (IDTP) is both significant and a challenge and has attracted much interest from pharmaceutical and biomedical researchers. Proteins are crucial drug targets and have been widely studied, and human proteins have also been for the identification of drug targets. Traditional procedures of drug target identification are limited by labour-intensive and time-consuming biomedical experiments [1,2], which tend to be performed within specific domains of research, leading to low efficiency and limited search scope. The low ratio of drug target proteins (DTPs) among human proteins also aggravates such conditions, and failed results are commonly due to poorly planned experiments that lack fine analysis. With the rapid development of new techniques for biochemical measurements and bioinformatics, additional informative characteristics about proteins are now available to researchers, thus providing novel approaches for biomedical tasks. Several plausible frameworks with foundations in data mining have been proposed to tackle these problems and are recognized as a key preclinical step in the drug discovery process [2,3]. Sufficiently mining task-beneficial information from protein characteristics would theoretically solve IDTP while avoiding expensive and redundant long-term experiments. The objective of this study was to provide a novel framework accompanied by bioinformatics analyses and machine learning techniques to guide IDTP and to ultimately recommend reliable DTPs for experimental validation by researchers in specific domains.

As discussed above, research on drug targets requires both designing experiments and identifying target and validation steps. Recommending reliable underlying drug targets from a given database plays a critical role in this research and represents the focus of this study. Two types of methodologies have dominated research: a systems perspective approach and a molecular approach [4,5].

In total, 218 molecular targets for approved drug substances were catalogued by [6], and 324 targets for approved therapeutic drugs in all classes were suggested by [7]. Recent trends towards the introduction of drugs that modulate previously unexploited targets were discussed in [8], which involved discussions of drug pharmacology networks. In [9], a bipartite graph was developed that established target connections between US Food and Drug Administration-approved drugs and proteins. Prediction methods for molecular targets based on aspects of chemical similarity in 2D structures were proposed in [10,11]. Phenotypic side effects were noted in [12], which the author used as an inference technique to confirm that a target was shared by two drugs. Proteins and nucleic acids represent the dominant proportion of drug targets, and the remarkable development of knowledge discovery in biochemistry, molecular biology and cell biology has accelerated the process of IDTP. In addition, a protein seldom acts alone but regulates other molecules to execute its function. With the application of high-throughput technologies to omics data, such as yeast two-hybrid protein interactions, researchers have additionally focused on methods based on protein-protein interactions (PPIs). An increasing number of PPI modulators have been detected and evaluated clinically [13]. PPIs offer intuitive information to systematically characterize how drug targets interact with the corresponding proteins.

The topology of the complex network of intercellular protein interactions may contribute to studies on target prediction [14]. As such, protein networks have been studied based on graph theory methods [15], and power graphs have been analysed to explicitly represent reoccurring network motifs. Drug-target protein networks and gene regulatory networks are systematically different from other networks and have been studied in interactive networks. High-throughput methods have also been applied to detect novel connections and to build many records of identified interactions [16]. Yamanishi et al. [17] summarized four types of drug-target interaction networks in which correlations among the similarity of drug structures, sequence similarity and the topology of the drug-target interaction network were revealed. All of these methodologies aim to employ time-specific or space-specific information for the identification tasks. Finally, several synergistic, time- or space-sensitive treatments considering the multidimensional use of drugs have been proposed with the assistance of these systems biology approaches [16].

In addition to the above two mainstream methodologies for IDTP, data mining techniques have been employed [5]. In this study, we have developed an improved and updated version of the data analysis methodology proposed by Bakheet and Doig [18] for determining the properties of drug targets (i.e., proteins targeted by drugs [19]) from the human proteome. Relevant previous works can be summarized as follows: Bakheet and Doig [18] built a support vector machine (SVM) with sequence information from 148 human DTPs and 3573 contaminated non-drug target proteins (NDTPs), in which a genetic algorithm was utilized for property selection. A machine learning tool (SMQQ) was designed to predict the distance deviation of each residue in a single protein model, and SVMs were trained with the sequence and structure properties of proteins [20]. Four novel stacked denoising auto-encoder-based SVMs were developed to predict the residue-specific quality of individual protein models in CASP11 [21]. Liu et al. [22] combined a stacked denoising auto-encoder with SVMs to predict the binary DNA methylation status of CpG sites and achieved improved performance. Former data mining approaches for IDTP have considered the task as a supervised binary classification problem, which may lead to severe outcomes. As the DrugBank database is continually renewed, some DTPs have been definitively confirmed, while others cannot be clearly recognized [23]. Even the non-drug target dataset may inevitably contain drugs that may later be shown to be drug targets [18]. Directly considering the non-DTPs as one class associated with uncertainty causes a failure to recall some DTPs. In light of such consequences, a novel framework is proposed for these conditions. Here, we utilize the sequence information of proteins to accomplish this task.

## Materials and methods

### Data collection and preprocessing

#### Data collection

**Fig 1:**
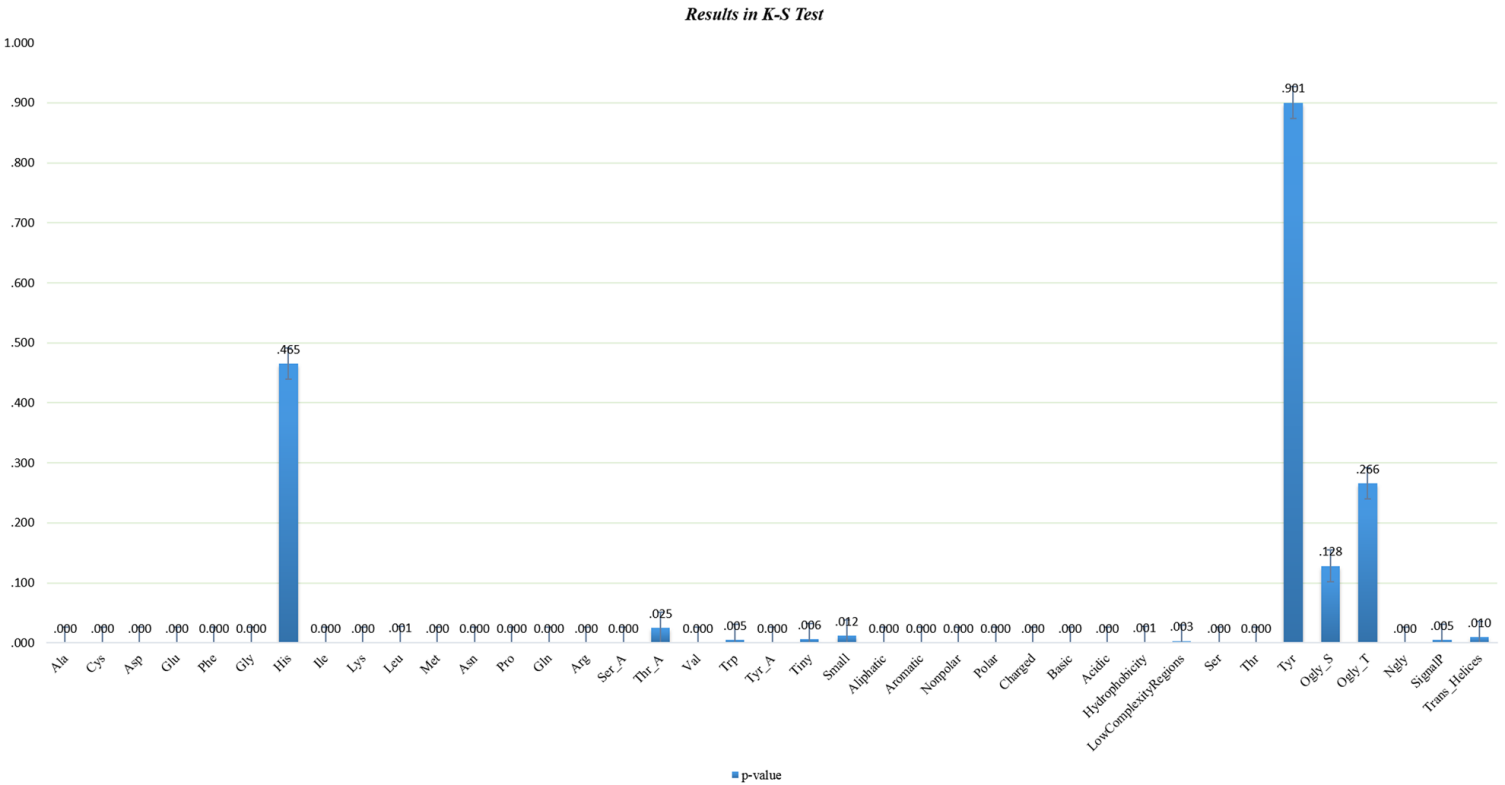
Results of the K-S Test. The p-values of the different properties of DTPs and contaminated NDTPs according to the Kolmogorov-Smirnov test.

As the main source of catalysts, signalling messengers and molecular machines in biological tissues [24], proteins interacts with each other to form the basis of signal transduction pathways and transcriptional regulatory networks. Target proteins are a family of functional biomolecules that are biologically controlled by active compounds. The collected dataset was obtained from the DrugBank database (Version 3.0), which includes 1604 proteins as drug targets [25]. Factors such as water solubility, hydrogen ion concentration (pH), bases and structure are strongly related to druggability. Thus, chemical or physical properties are both decisive and fundamental in determining whether a protein is a potential drug target. Here, some manipulations were performed to extract properties according to [18], with a focus on employing redundancy information to collect DTPs and contaminated NDTPs. The whole process was as follows. Since amino acids play a crucial role in determining the biological activity of proteins, the statistics of the protein properties were calculated using pepstats, an online software from EMBOSS [26]. Amino acid properties including tiny, small, aromatic, aliphatic, polar, non-polar, charged, and basic were calculated [18]. In addition, we extracted properties such as single peptide cleavages [27], transmembrane helices [28], low complexity regions [29], N-glycosylation [30], and O-glycosylation [31]. In total, 39 properties were employed for the identification task, of which 31 properties were continuous, and the remainder were nominal. More detailed information about the properties can be found at http://pan.baidu.com/s/1jINqbAY. Then, protein sequences with identities greater than 20% were removed by PISCES [32]. Finally, a dataset with 517 DTPs and 5376 contaminated NDTPs was obtained. The 5376 contaminated NDTPs represented the test dataset from which the potential DTPs were predicted. That is, our work was inspired by [18], but we expanded the testing dataset from 3573 to 5376 contaminated NDTPs. To illustrate the effectiveness of the properties, a Kolmogorov-Smirnov test was employed to identify differences in individual properties between the two classes. As shown in Fig 1, only four selected properties were not significantly different at the level of 0.05 in the distribution of the two classes; thus, our task would benefit from the extraction of these properties. Please refer to the supporting materials S1 File for more detailed information regarding the dataset.

#### Data preprocessing

The chemical and physical properties extracted were rather sparse, comprising a mixture of real valued continuous properties and nominal properties. We preprocessed the properties such that all continuous properties were normalized, and nominal properties were transformed using one-hot encoding.

Normalization for the continuous properties was performed as follows:

$$z^{i}=\frac{x^{i}-\mu }{\sigma }$$

where z^i^ is the normalized value of *x*^*i*^, *μ* is the mean of the population, and *σ* is the standard deviation of the property.

One-hot encoding is commonly used to code categorical properties. Categorical properties are attribute-value pairs in which the value is restricted to a list of discrete possibilities without ordering. In our research, some of the collected properties were not continuous, and thus one-hot encoding was employed for the initial representation. Specifically, for a property with d states, the representation of this property can be encoded in a d-dimensional bit vector.

After normalization and one-hot encoding, the dimension of the properties increased to 283 with some properties overlapping in the representation.

### Proposed framework

**Fig 2:**
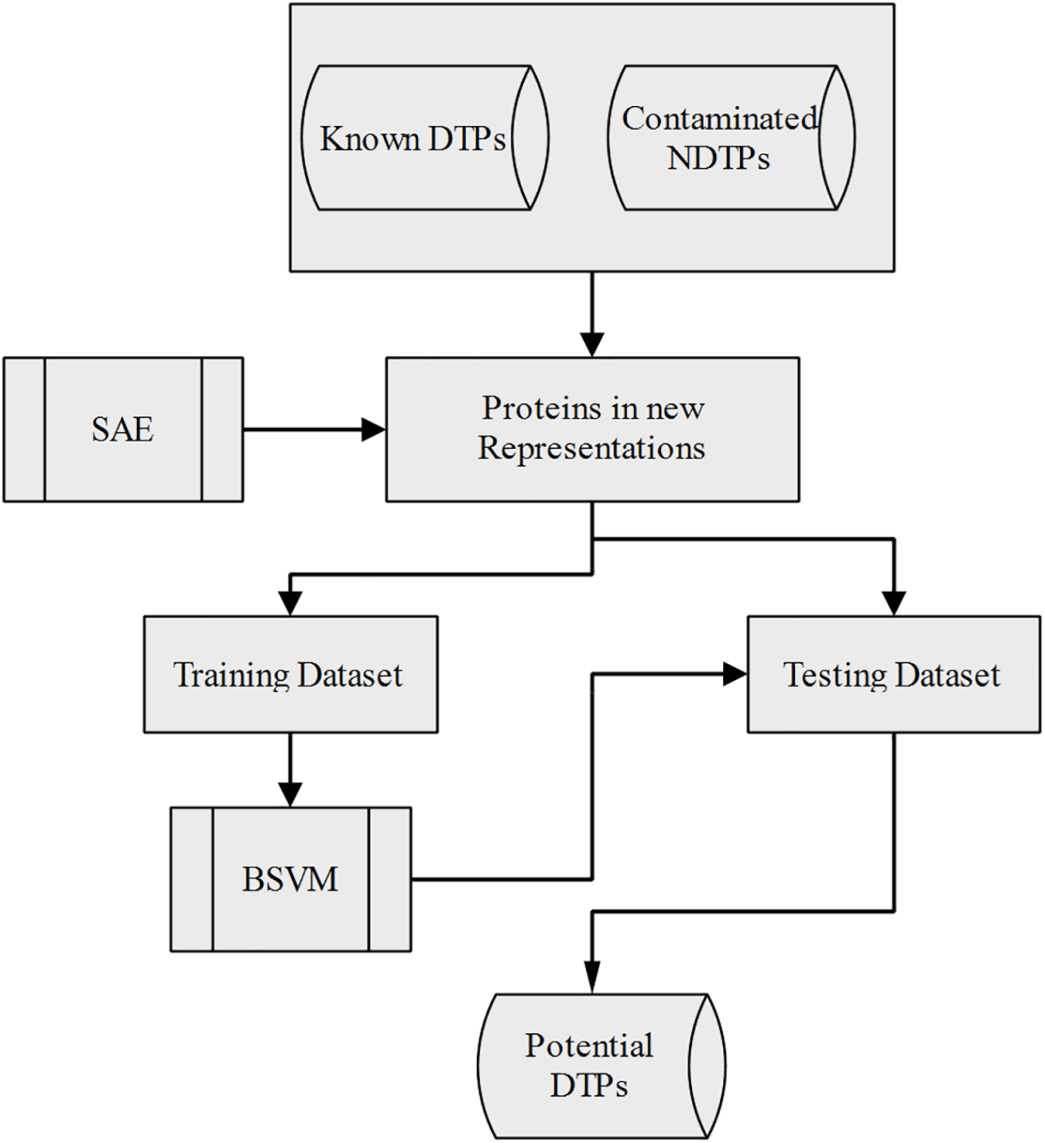
Flowchart of the proposed framework.

To take advantage of the efficient representations provided by deep learning models, we initially trained stacked auto-encoders (SAEs) to extract properties from the original protein representations, and then a typical cost-sensitive-based positive and unlabelled (PU) learning algorithm biased support vector machine (BSVM) was implemented for the identification task. **Fig 2** gives the detailed information of identification process. This novel framework illustrates the potential capability for IDTP, as described in the Results and Discussion.

#### Deep learning and feature representation

Increasing attention from both industry and academia has rapidly promoted deep learning models. They provide great power for feature representation and outstanding performance for several tasks. Deep learning models have dominated a number of tasks, such as voice detection [33], handwritten number recognition [34,35], image classification [36], and statistical machine translation [37], and most of these deep learning models are recognized as state-of-the-art models in their respective domains. Moreover, some researchers have employed such models to overcome difficult problems in bioinformatics [38–40].

Inspired by the representation power of deep learning models, SAE was introduced to improve the extraction of protein properties as described below.

**Fig 3:**
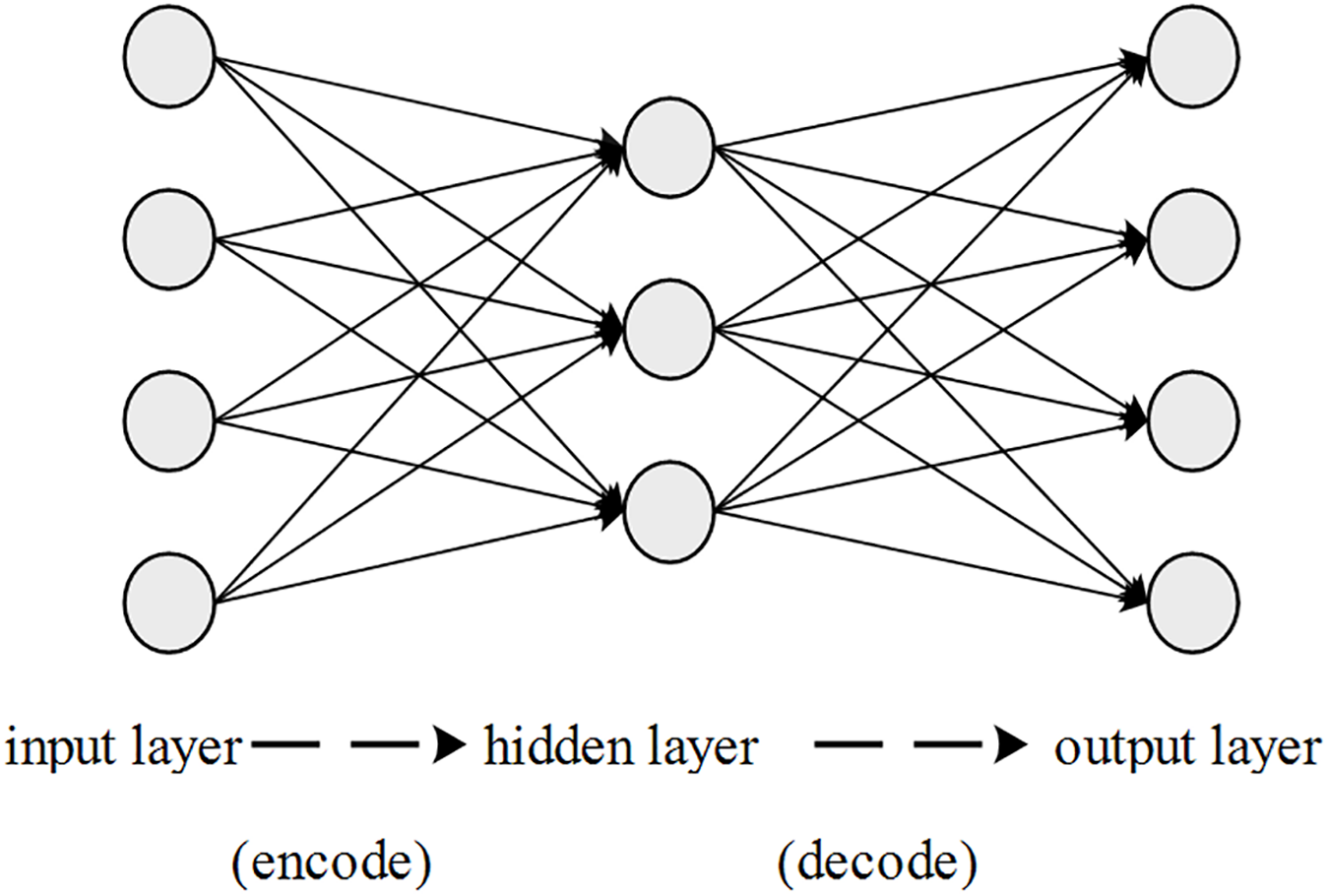
Structure of the auto-encoders.

The auto-encoder (AE) is the elementary unit of SAE and can be described by a three-layer neural network, as shown in **Fig 3**. The AE was previously applied for data compression in [41], and further information can be found in [42,43]. Only one hidden layer is employed in the AE to provide an intermediate representation. An AE can be described as follows from a mapping perspective.

Given an original vector of input x, the intermediate representation is acquired via a linear transformation between the input layer and the hidden layer and non-linear activation in the hidden layer. The derived hidden representation is

y=f(W*x+b)

where f corresponds to the activation function, W is the matrix of the linear transformation, and b is the bias term.

After the encoding process, decoding is run by a map g.

$$z=g(W^{∼}*y+b^{∼})$$

Because the goal of the AE is to capture latent factors for reconstruction of the input, squared error is frequently used, as follows.

$$L(x,z)={\left|\left|x-z\right|\right|}_{2}^{2}$$

If the input is represented in binary code, the cross entropy can also be calculated.

$$L(x,z)=-\sum_{i=1}^{m}(x_{i}lnz_{i}+(1-x_{i})\;\ln\left(1-z_{i}\right))$$

**Fig 4:**
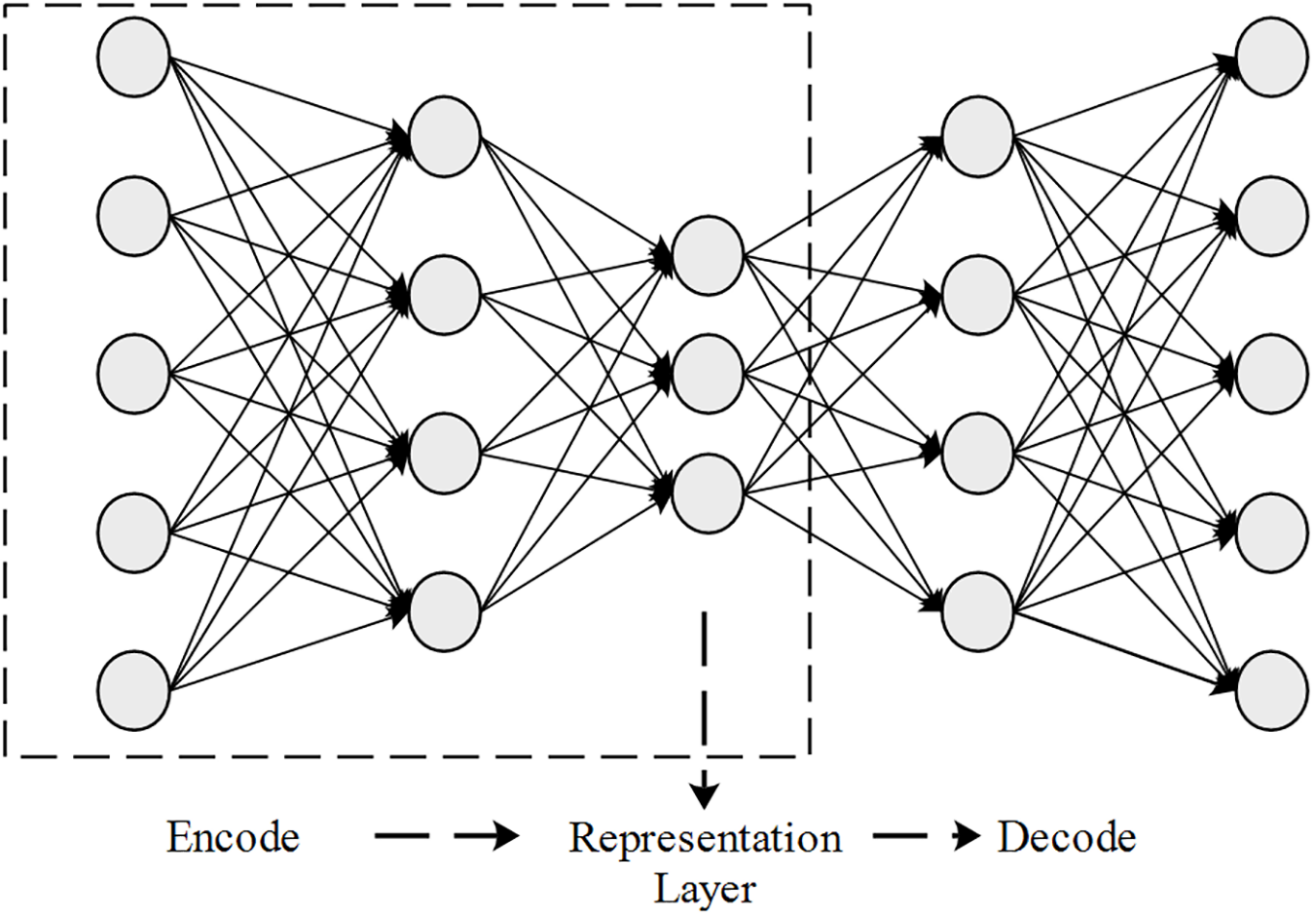
Structure of the stacked auto-encoders.

By stacking AEs layer-by-layer such that the output of the AE serves as the input of the next AE, a deep network can be generated as an SAE. The parameters learned in the SAE can then be exploited to initialize the deep neural network as a pre-training process. However, in this study, we exploit the capability of SAE in representation learning to use the output of the intermediate layer as the new feature representation. **Fig 4** describes the structure of SAEs.

#### PU learning and BSVM

Motivated by a soft margin SVM, BSVM was introduced by Liu et al. [44] when tackling text categorization with positive and unlabelled available data.

Given the training set {(x_1_,*y*_1_),(*x*_2_,*y*_2_),..,(*x*_*n*_,*y*_*n*_)|*y*_*i*_ = 1 *or* − 1} in which the first k samples are positive {y_i_ = 1|*i* = 1,2,..,*k*} and the remaining samples are unlabelled, we concentrate on precisely retrieving positive samples from the unlabelled set in our specific retrieval task.

Under such circumstances, the following hypothesis is reasonable. Unlabelled samples are generated such that the negative samples are contaminated by a small proportion of positive samples. Based on probability, the distribution of the unlabelled samples, h_u_, is generated from a mixture of the distributions of the positive samples, *h*_+_, and negative samples, *h*_−_.

$$h_{u}(x)=\alpha h_{+}(x)+(1-\alpha )h_{-}(x)$$

The conclusion is drawn from the hypothesis that the probability distribution of the unlabelled samples closely approximates the negative samples when the contaminating proportion ratio α is sufficiently small:

$$\frac{h_{u}(x)}{h_{-}(x)}=\frac{\alpha h_{+}(x)}{h_{-}(x)}+(1-\alpha )$$

Based on this assumption, the unlabelled samples are directly considered as negative samples during the classification process.

The BSVM can be modelled as follows:

$$\min\frac{1}{2}w^{T}*w+C_{+}\sum_{i=1}^{k}\zeta_{i}+C_{-}\sum_{i=k+1}^{n}\zeta_{i}$$

$$s.t\;y_{i}(w^{T}*\phi (x_{i})+b)\geq 1-\zeta_{i}$$

$$\zeta_{i}\geq 0,i=1,2,..,n$$

where the hyper-parameters *C*_+_ and *C*_−_ control the penalization of samples for violating the corresponding support hyper-planes. Intuitively, the weights of the penalizing slacked variables of the two types, *C*_+_ and *C*_−_, should be finely adjusted.

A heuristic implementation for tuning the parameters *C*_+_ and *C*_−_ is to impose the constraint

$$C_{+}|P|=C_{-}|N|$$

where |*P*| and |*N*| represent the number of DTPs and NDTPs, respectively, to reduce the scope of parameter searching.

In summary, the novel framework takes the representations of proteins that are learned in the SAE as the new input for the BSVM to improve the final performance.

## Results and discussion

### Settings for SAE, BSVM and models for comparison

A better feature representation should both minimize information redundancy and capture properties more relevant to a specific task. SAE tends to satisfy both; however, it is also widely acknowledged that the tuning of parameters in deep learning models is a non-trivial and overwhelming task. Here, Keras [45], an easily implemented popular deep learning module, was used to establish and train the SAE model. The entire dataset of proteins was involved in training the SAE. To prevent over-fitting, the dataset was partitioned into two parts: 70% for SAE training and 30% for validation. The SAE training process was stopped early if the reconstruction error on the validation set began to markedly increase.

For the BSVM, we created stratified partitions of the DTPs and NDTPs in the new representation with 70% for training and 30% for testing. The BSVM was based on the modulation of the SVM model in scikit-learn [46]. In terms of structural implementation of the SAE, including the number of layers and the corresponding numbers of units, we performed several trials to obtain the best corresponding performance, and a 5-layer SAE implementation was finally selected. Various optimal hyper-parameters were searched in a grid, and the final results are listed in **Tables 1 and 2**. The final structure of our SAE was composed of five layers corresponding to 283, 140, 10, 140, and 283 units in layers one to five, respectively. The proteins were transformed through 10 dimensions using the SAE.

**Table 1:** Parameter settings in the SAE.

Parameters used for SAE training	
nb_epoch	100
batch_size	100
optimizer	adadelta
loss	mean_square
training_ratio	70%
validation_ratio	30%

**Table 2:** The optimal parameters for BSVM (SAE), BSVM (Wrapper), and BSVM (Origin).

Parameters	BSVM(SAE)	BSVM(Wrapper)	BSVM(Origin)
**gamma**	8.5	5	9.503
**c+**	47.17	4.5	8.552
**c-**	5.24	0.5	0.95

To verify the effectiveness of the SAE, a BSVM trained with the proteins of the original representations were also included in the experiments. Another state-of-the-art technique for feature selection, a wrapper method [47], was also adopted for comparison to the proposed model.

### Evaluation criteria

For the binary classification, the confusion matrix can intuitively evaluate the performance of the model. In the evaluation process, we insist the recall ratio of DTPs and the precision of NDTPs are both worthwhile to analyse.

In our IDTP task, the negative samples (NDTPs) dominate the dataset. The accuracy of the model does not provide meaningful performance, thus providing evidence of the imbalance. That is, traditional supervised models tend to identify most samples as the majority to over-focus the accuracy, thus hindering recall of the minority. As a trade-off between the recall ratio and precision, an F-score is introduced as an appropriate metric for performance. TP and TN represent the total correctly classified positive and negative samples, respectively, while FN and FP represent the number of positive and negative samples misclassified in the model, respectively. The F-score is a weighted average of the precision and recall ratios, given as follows.

$$F_{\beta }=\frac{\left(1+\beta^{2})(\frac{TP}{TP+FP}*\frac{TP}{TP+FN}\right)}{\frac{\beta^{2}TP}{TP+FP}+\frac{TP}{TP+FN}}$$

Here, we choose the F-score of the DTPs for evaluation with β = 1.

### Results and analysis

To eliminate the experimental result bias from randomness and to further validate the generalization of the models, we independently ran 10 iterations of the same BSVM experiment in which the proteins were partitioned 10 times for training and testing. For each iteration, the BSVM was trained using a different set of 70% of the proteins acquired from the random stratified partition, and the remaining 30% of proteins were used for testing. The parameters of the BSVM were chosen by performing a grid search using a range of parameters with the criterion of a maximum F1 score in the training set. For comparison, we trained another BSVM with parameters selected from the same range as above according to the average F1 score in a 5-fold cross validation and using the same training set as the original representation in the iteration. To evaluate the performance in detail, we computed the precisions of the NDTPs, the F1 score and the recall ratio of DTPs for each iteration.

**Table 3** shows the statistical results for the 10 iterations. Here, SAE-BSVM, Wrapper-BSVM and Origin-BSVM represent the BSVMs trained with the proteins of SAE embedded representations, wrapper extracted properties and the proteins of the original representation, respectively. According to the table, the three models behaved consistently in that all metrics of the training set were higher than those of the testing set. In addition, Origin-BSVM outperformed the other BSVMs in three metrics; however, the opposite result was observed for the testing set. These findings represent extreme circumstances in machine learning. One explanation is that the BSVM trained using the proteins of the original representation is at risk of severe over-fitting, especially in cases of class imbalance with the precision of the DTPs neglected. Another factor is the inability to extract discriminative information from the original representation. Such a problem is not encountered for the BSVM trained using the proteins from the SAE embedded representation or the wrapper technique. Regarding the testing results that actually reflect generalization capability, the SAE-BSVM was superior to the Wrapper-BSVM based on the F1 scores and recall ratios. Although the Wrapper-BSVM achieved the highest average precision of NDTPs of approximately 0.923, the recall ratio of the DTPs is the most important parameter. In addition, because some DTPs are involved in NDTPs, the precision of the NDTPs is not necessarily high in practical applications.

**Table 3:** Statistical results of the average of 10 iterations of the three models. Figures in parentheses are the corresponding variance of the 10 independent results.

	Dataset	F1 score-DTPs	Recall Ratio-DTPs	Precision-NDTPs
**SAE-BSVM**	**Training**	0.349(0.179)	**1(0)**	**1(0)**
	**Testing**	**0.234(0.013)**	**0.712(0.101)**	0.587(0.027)
**Wrapper-BSVM**	**Training**	0.179(0.087)	0.482(0.131)	0.926(0.006)
	**Testing**	0.169(0.016)	0.451(0.141)	**0.923(0.006)**
**Origin-BSVM**	**Training**	**1(0)**	**1(0)**	**1(0)**
	**Testing**	0(0)	0(0)	0.91(0)

**Fig 5:**
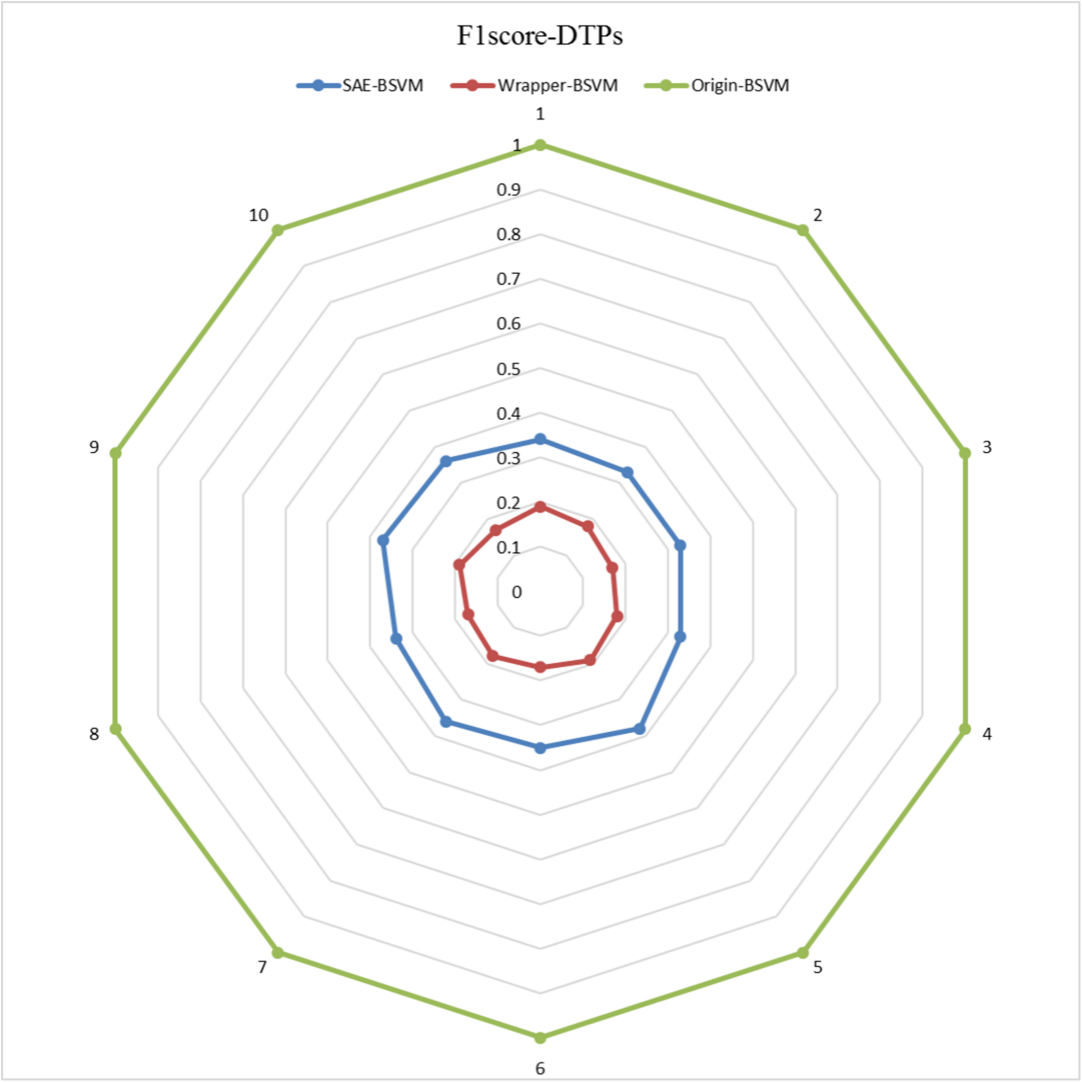
The F1 scores of DTPs in 10 independent iterations for the training dataset.

**Fig 6:**
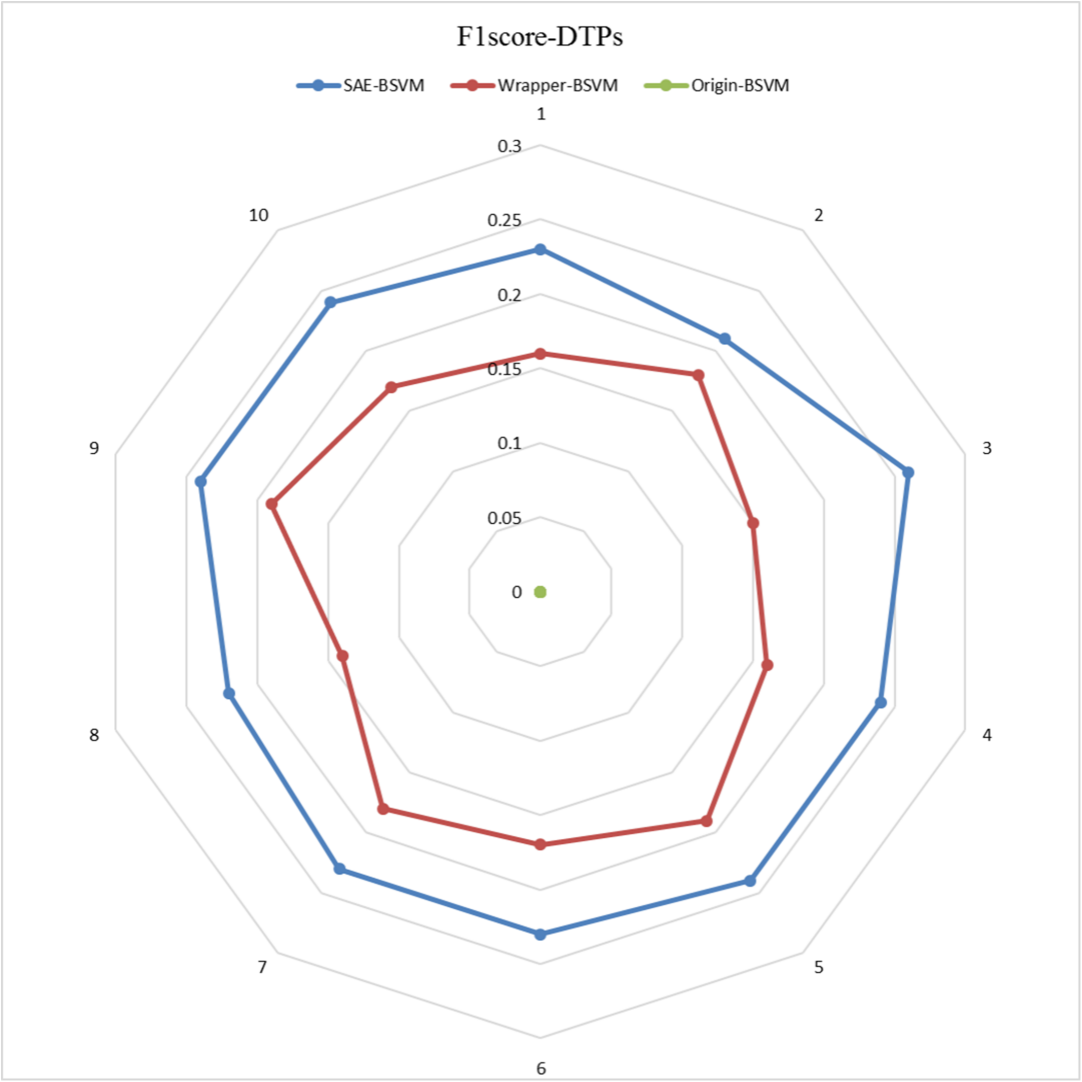
The F1 scores of DTPs in 10 independent iterations for the testing dataset.

**Fig 7:**
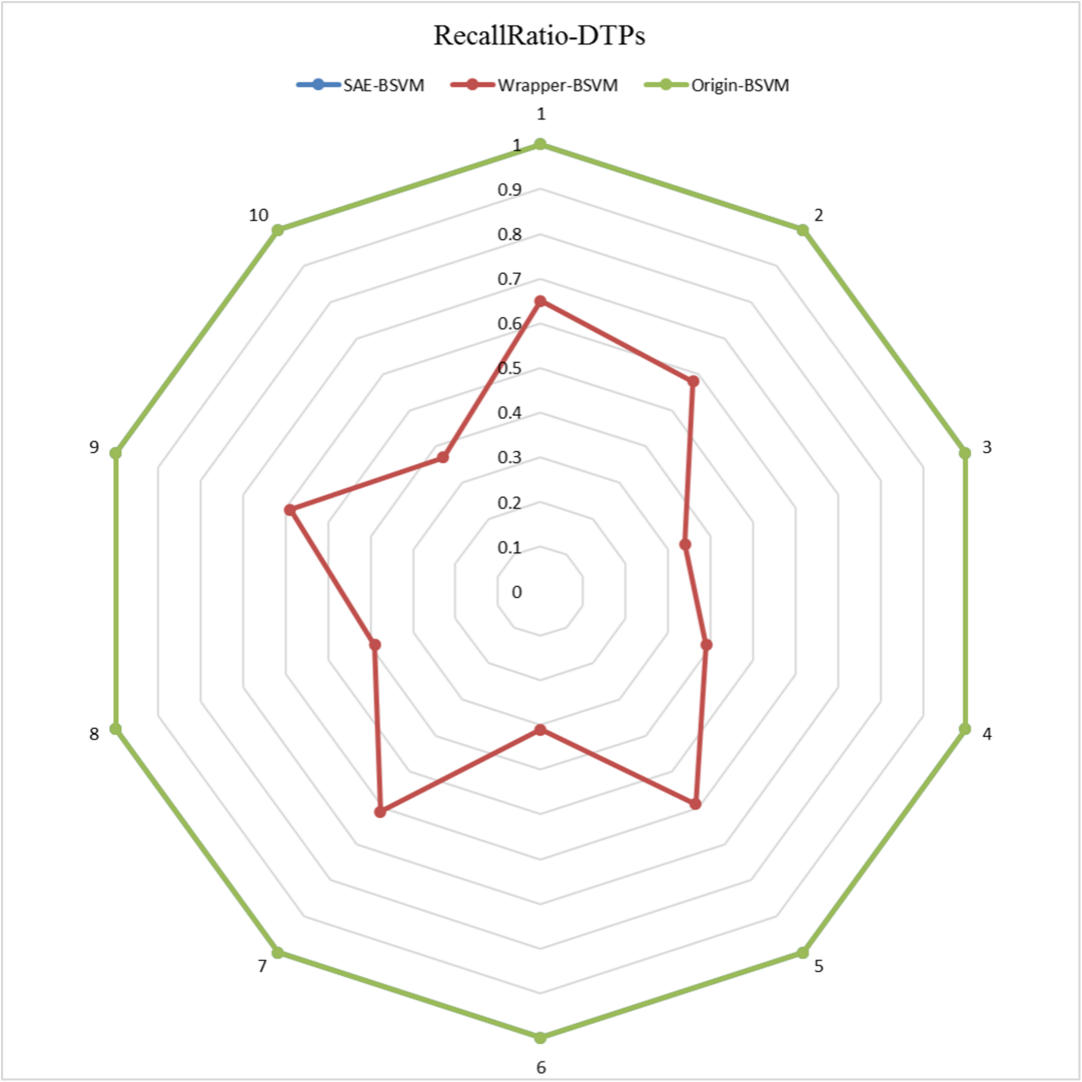
The Recall Ratios of DTPs in 10 independent iterations for the training dataset.

**Fig 8:**
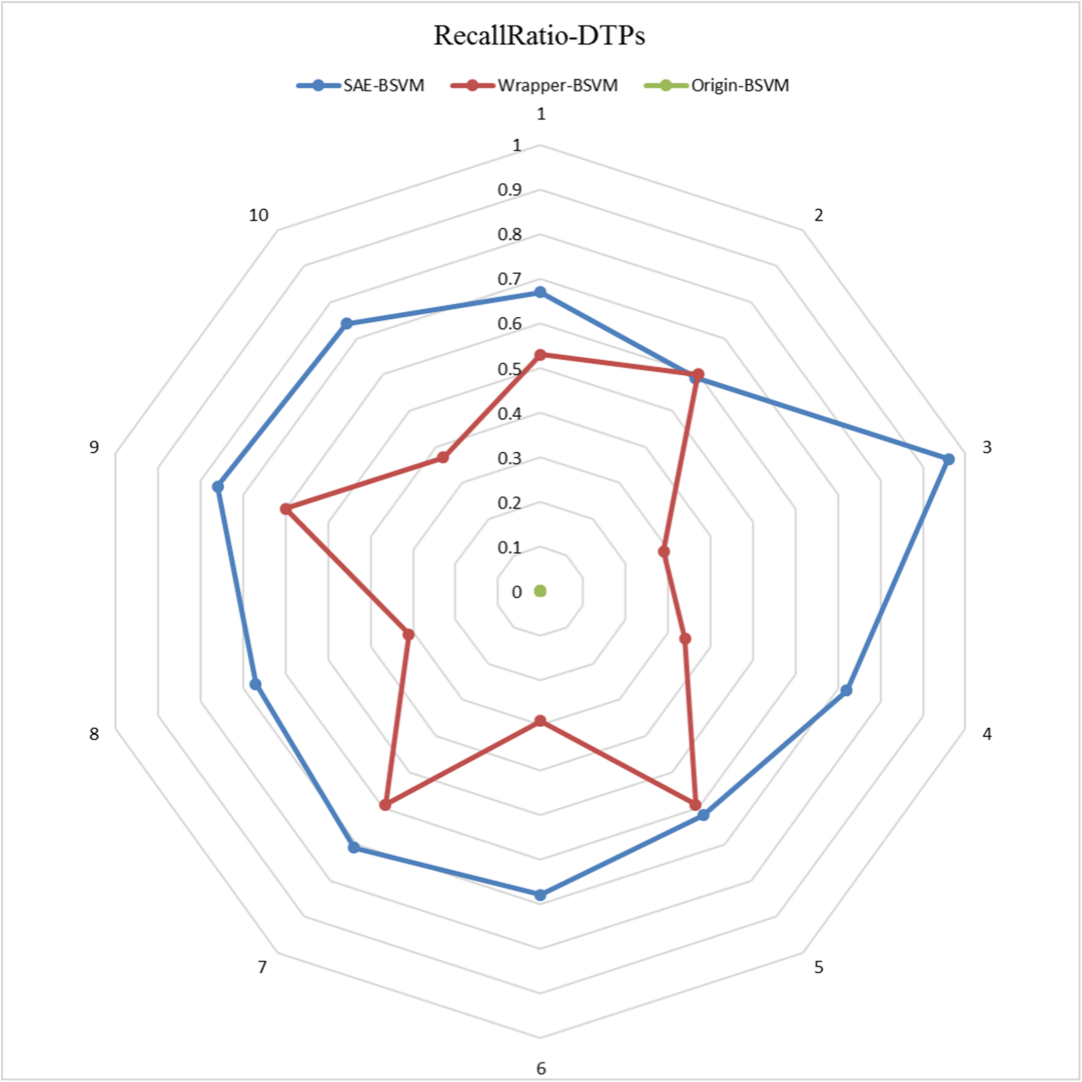
The Recall Ratios of DTPs in 10 independent iterations for the testing dataset.

**Fig 9:**
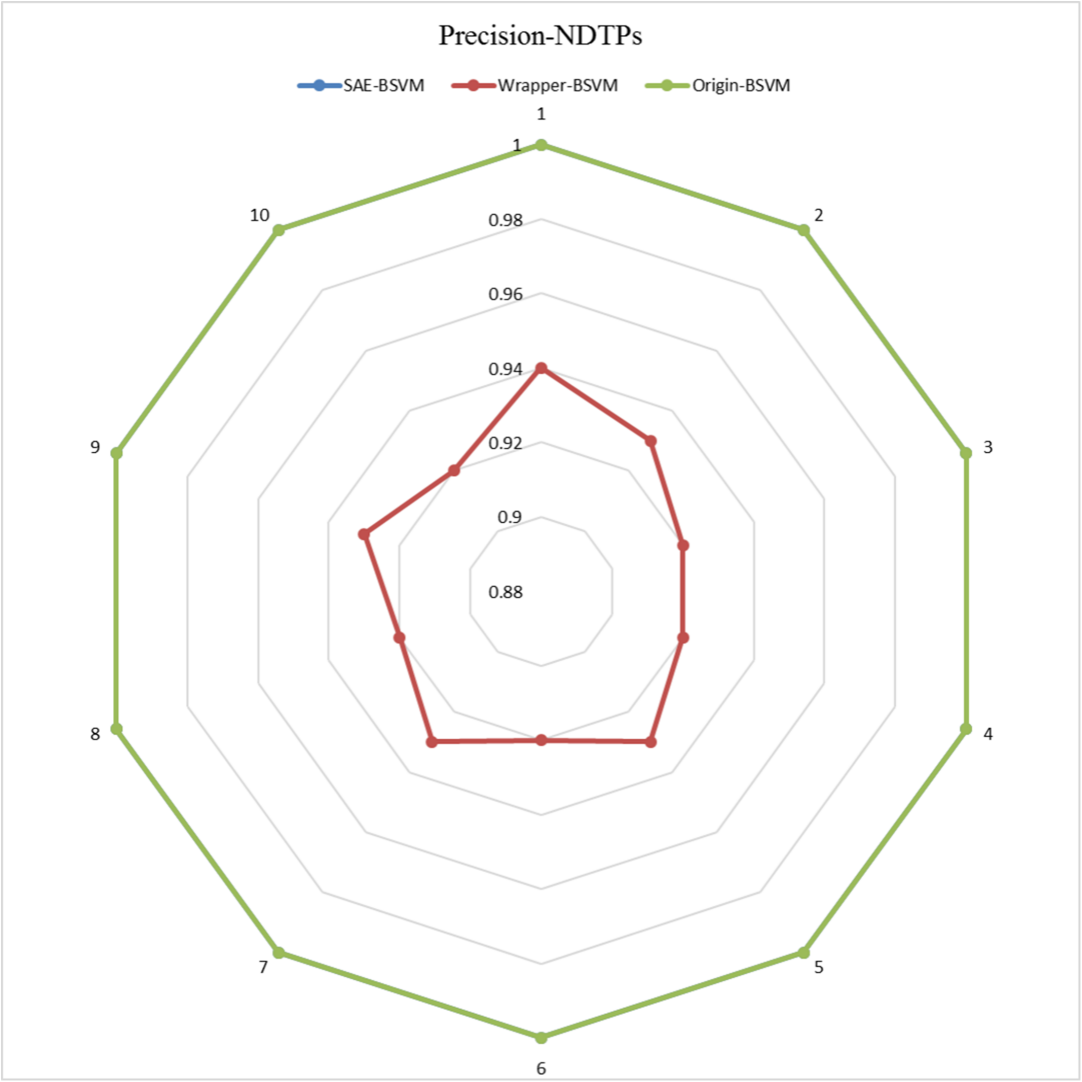
The Precisions of NDTPs in 10 independent iterations for the training dataset.

**Fig 10:**
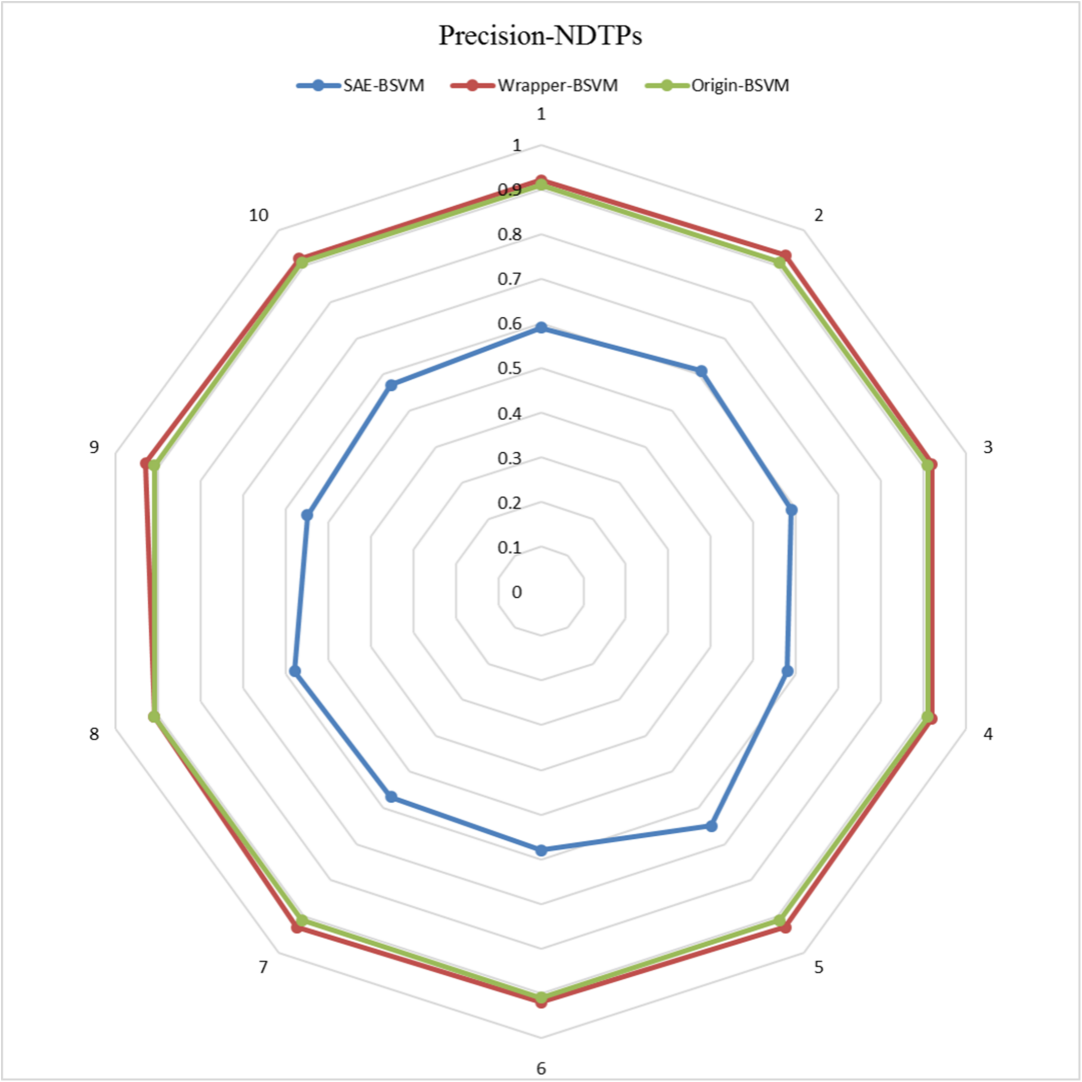
The Precisions of NDTPs in 10 independent iterations for the testing dataset.

The results of the 10 experiments are illustrated in the six radar plots corresponding to the three metrics as **Figs 5–10**. As shown in the figure, the stable performance of our method is represented by blue curves for the three metrics. Another notable phenomenon illustrated in **Figs 6** and **8** is that the testing results for Original-BSVM were consistently 0 in both F1 score and recall ratio of DTPs. This result may be due to dimension disaster of Original-BSVM based on the original properties, which were high in dimension and not sufficiently discriminative. In addition, the imbalanced distribution of DTPs and contaminated NDTPs led to a poorer recall ratio of DTPs, with a value of 0, and thus the F1 score remained 0 as well.

The above analysis suggests that the SAE functions well to provide a robust representation method and to prevent over-fitting.

In the final process to retrieve the DTPs from the NDTPs, potential DTPs were derived by merging the NDTPs identified as DTPs in the training process with those identified in the testing process. We obtained a total of 1285 doubtful DTPs using SAE-BSVM on the NDTPs in one iteration. Thus, approximately 23% of the NDTPs were possible DTPs worthy of further study in biomedical experiments. Since the identification was all of our concern in the research and it was worthwhile to provide a list of identified doubtful DTPs for the further validation, we established a website link http://pan.baidu.com/s/1dFrC2yP to store the list of recommended doubtful DTPs. Such website served the pharmaceutical experts for carrying out further experiments. It should be emphasized that the prediction results vary with each iteration due to the randomness in both the SAE training process and sampling process, but the results for the proportion of possible DTPs were stable, at approximately 23%. Such result was exactly the same with the proportion of predicted DTPs which shared target-like properties in the former research [18]. Further analysis on the results were as follows. For one thing, the fact that both of the research achieved the same result on the proportion of predicted likely DTPs has confirmed the proportion [18] again though the two ways of properties’ processing were employed differently in the original work and ours. For another thing, an updated version of protein database was utilized in our experiment, so the number of predicted likely DTPs was enlarged in comparison with the former work [18]. In total, the conclusions of both research shared the consensus in the plausibility of experiment framework using SVM with chemical and physical properties of proteins.

## Conclusions

We designed a novel framework for IDTP in which an SAE was first adopted as the feature representation technique. Compared with the results of Origin-BSVM and Wrapper-BSVM, the SAE embedded properties prevented over-fitting and enhanced generalization. The BSVM that originated from the PU learning task was also used as the classifier, which reduced imbalanced distribution effects. Finally, our framework identified approximately 23% of proteins among the original NDTPs as possible DTPs. Future studies of IDTP are needed, and semi-supervised learning methods should be explored to increase performance.

## Supporting information

S1 File Properties of the proteins in our experiments. Please refer to the Supporting Information.
(XLSX)
